# Differential changes in the morphology and fuel loads of obligatory and partial migrant passerines over half a century in Britain

**DOI:** 10.1186/s40462-024-00497-3

**Published:** 2024-09-02

**Authors:** Holly R. W. Pickett, Robert A. Robinson, Robert L. Nudds

**Affiliations:** 1 School of Biological Sciences, Faculty of Biology, Medicine and Health, University of Manchester, Oxford Rd, Manchester, M13 9PL UK; 2 British Trust for Ornithology, The Nunnery, Thetford, Norfolk, IP24 2PU UK

## Abstract

Migratory distances and stopover locations are changing for many passerines in response to climate change. Morphological changes have been linked to rising global temperatures in both migrants and residents, but the implications of these changes on fuel loads, and associated flight ranges are little studied. Wing length and body mass changes between 1964 and 2020 were calculated for 15 migrant and partially migrant passerines in Britain. Changes in fuel load and lean body mass were also estimated and used to predict flight ranges. Twelve of the species have undergone morphological changes and eight species, estimated fuel load changes. Nine species were estimated to have reduced flight ranges, indicating that the morphological changes have not compensated fully for the reduction in flight range experienced since 1964. Partial migrants showed greater decreases in flight ranges than did full migrants, which may indicate greater behavioural plasticity in the former. Those species which do not adapt morphologically or behaviourally may be unable to complete long migrations, resulting in restriction to sub-optimal breeding/wintering habitats, or a need for a sooner first stop and more stops en route. This highlights the importance of conserving migratory stopover sites, particularly in the Mediterranean and North Africa that immediately precede major geographical barriers, as-well-as breeding and wintering grounds.

**Supplementary Information:**

The online version contains supplementary material available at 10.1186/s40462-024-00497-3.

## Introduction

Climate change, primarily driven through a global warming [1], is a leading cause of bird population decline with major impacts on geographical range, behaviour and population dynamics [2]. Migratory species are likely to be most profoundly affected as they must adapt to changes in these variables at breeding and wintering grounds as well as on migration. There is extensive evidence of changing passerine migration phenology in response to climate change. Some long-distance migrants are advancing autumn migration enabling them to reach the Sahel zone while it is still in its rainy season [3, 4]. Others are prolonging the autumn migration period or delaying migration [5, 6]. Prolonged migration is particularly interesting as it could be tied to length of time spent at stop-overs or number of stops. Additionally, milder conditions in the north are allowing short-distance migrants to stay longer at breeding sites before migrating [4]. As a result, it is thought that larger portions of partially migrant populations may become resident while the proportion of migrant species in northern communities may decrease, though empirical evidence of this is inconclusive [7, 8].

Climate change is also affecting the distances birds need to migrate. Climate niche modelling predicts a northerly shift in breeding ranges, indicating that some species may experience longer migrations [9–11]. Estimated southerly range shifts are more variable among species, but most studies also predict poleward shifts in winter ranges, again potentially lengthening migration distances [12]. The Mediterranean basin is also expected to warm, which could allow winter ranges to spread into higher altitudes for shorter-distance migrants. Moreover, the drying of the southern Mediterranean could mean these species do not travel so far south, reducing migration distances [13]. The effect of a poleward shift in wintering range is dependent upon whether a northern hemisphere breeding species winters in the Northern or Southern hemisphere. The former will experience a decreased migration distance and the latter a lengthened migration distance.

These changes in migratory phenology, behaviour, and routes could impose new selection pressures on associated morphology. It is well established that wing morphology is linked to migratory strategy. Migrants tend to have a higher aspect ratio and more pointed wings than residents [14, 15]. For example, in partially migratory *Sylvia atricapilla* populations, migratory individuals have longer, more pointed wings [16] and *Oenanthe* species that migrate further also have longer, narrower (a higher aspect ratio) wings than shorter migratory-distance species [17]. Hence, climate driven changes in migration distance and behaviour are likely to result in changes in wing morphology. For example, North American migratory birds are decreasing in body size concomitantly with increasing summer temperatures, whilst increasing their wing lengths, perhaps as a compensatory adaptation to body size induced changes to flight metabolic costs [18]. The changes induced by climate change are likely to differ among species, not least because the rate of morphological change is thought to increase concomitantly with decreasing body size [19]. Some studies have linked passerine morphology to climate [20, 21] through the temperature driven mechanism defined by Bergmann’s rule [22], which posits that individuals should get structurally smaller as ambient temperatures rise. Bergmann’s rule alone, however, should be treated with caution when interpreting long-term changes in morphology, as advanced migration can expose birds to lower temperatures in breeding areas and possibly mitigate the effects of climate on body size [23].

Another key metric which may be affected by climate change is migratory fuel load (*M*_f_). Spring arrival fuel load depends on habitat quality in stopover areas south of the Sahara [24], which, along with the Mediterranean Sea, represents a significant ecological barrier for migrating Palaearctic birds [25]. Furthermore, fuel deposition during autumn migration depends on food availability in the Mediterranean region just before the Sahara crossing [26]. Birds with larger fuel loads on departures from stopovers are more likely to make direct, cross-sea migrations, while leaner individuals are more likely to migrate along coastlines [27]. With this knowledge, it is clear that climate driven changes to *M*_f_ could drastically impact migration behaviour, while departure fuel loads from breeding grounds and stopovers will likely affect the success of the journey. However, little is known about if/how autumn departure *M*_f_ are changing and the impacts this could have. Pre-migratory fuel loading, both after breeding and at stopover sites, could be affected in several ways, not least of which is a temporal uncoupling of food requirements and availability [28, 29]. Fuel loading may also be affected if climate-driven morphological or behavioural changes alter species’ fuel requirements.

It has been suggested that migrant passerines are undergoing reductions in non-stop flight range, which would require an increase in the number of stopovers required to complete the migration distance, but these range calculations assumed a fixed fuel load of 30% [30]. Birds, however, may compensate for an increased need to stopover by increasing their fuel load, or climate changes may impact fuel load deposition, so the assumption of a 30% fuel load may not be robust. Clearly, knowledge of the factors that influence migratory fuel loads is critical in interpreting how climate change might impact species with differing migratory strategies. The primary question is how changes to birds’ fuel load may affect their flight range. Climate-driven impacts on feeding, including altered food availability and weather conditions, will also affect how frequently and where individuals stop en route [30]. With threats of habitat change and hunting at traditional stopovers in the Mediterranean, estimations of how a species’ stopover behaviour might change could help inform conservation efforts at these sites [31].

Here, changes in the morphology of 15 species of migrant passerines over half a century are investigated, using long-term data provided by 3 British bird observatories. We used the only avaliable predictive formulae [32] for measuring fuel load (*M*_f_) and lean body mass (*M*_b−lean_) to pursue a novel way of estimating changes in *M*_f_ upon autumn departure from the UK. The mass of fat (*M*_fat_) obtained from the formulae [32] is combined with biometrics to estimate changes in the distance these species can fly before needing to refuel. *M*_f_ is defined as *M*_fat_ expressed as a percentage of *M*_b_. Further comparisons of biometrics, *M*_f_ and flight ranges are made between partial and full (obligatory) migrants. Irrespective of the mechanism of the impact of climate change on migratory behaviour, for example, thermoregulation, changing phenology or length of migration period [5, 30], the previously observed morphological change [18, 19] that accompanies climate change must affect flight performance and therefore impact migration. Furthermore, morphological change will also likely impact *M*_f_, which will influence migratory distance capabilities and effects on *M*_f_ have yet to be considered. Accordingly, we tested the hypothesis that passerine morphology has changed between 1964 and 2020, a period in which average temperatures have increased by 0.98ºC (NOAA 2021 [33]), then quantified how such changes could affect migratory fuel load and flight range.

## Methods

### Study species and sites

15 passerine species that were both migratory and had extensive handling records were selected for the analyses and divided into full and partial migrants (Table 1). *Turdus iliacus* is a winter migrant to the UK, so was excluded from *M*_b_, *M*_f_ and range comparisons by migratory strategy, because these would be based upon arrival *M*_f_ rather than departure *M*_f_ and therefore not comparable with those of the other departing migrants. *T. iliacus* was, however, included in comparisons of lean body mass (*M*_b−lean_) and wing length (*l*_wing_) between full and partial migrants, as a full migrant, as these variables should not be impacted by depleted *M*_f_ during migration so remain comparable.

**Table 1:** The results of linear regression of wing length (*l*_*wing*_) against year by species, including changes between 1964 and 2020 and mean *l*_*wing*_ for species which did not show a significant change

Species	Migratory Strategy	Gradient (*m*)	Intercept (*b*)	Adjusted *R*²	Standard error of residuals	*F*	*P*	Mean *l*_wing_ (mm)	Δ *l*_wing_ (%)	
***Atricapilla pratensis***	Full	0.020	39.38	< 0.01	3.18	3.04	0.08	80.11	1.43	
***Erithacus rubecula***	Partial	0.004	65.67	< 0.01	2.02	0.58	0.44	73.2	0.29	
***Oenanthe oenanthe***	Full	**0.171**	**-238.63**	**0.32**	**4.35**	**22.94**	**< 0.01**		**9.91**	
***Phylloscopus collybita***	Full	0.005	50.59	< 0.01	3.01	0.22	0.63	59.75	0.43	
Prunella modularis*	Partial	**0.029**	**12.05**	**< 0.01**	**2.12**	**45.21**	**< 0.01**		**2.35**	
***Phoenicurus phoenicurus***	Full	-0.012	103.42	0.00	2.40	1.69	0.16	78.76	-0.88	
***Phylloscopus trochilus***	Full	**0.026**	**13.69**	**0.02**	**3.00**	**34.14**	**< 0.01**		**2.25**	
Sylvia atricapilla*	Full	**0.023**	**28.02**	**< 0.05**	**2.02**	**28.17**	**< 0.01**		**1.77**	
Sylvia borin*	Full	0.004	69.46	< 0.01	1.83	0.35	0.06	77.49	0.29	
Curruca communis*	Full	**0.044**	**-16.43**	**0.09**	**2.02**	**189.30**	**< 0.01**		**3.53**	
***Sturnus vulgaris***	Partial	**0.062**	**6.31**	**0.06**	**3.68**	**8.61**	**< 0.01**		**2.71**	
***Turdus iliacus***	Full (winter)	**0.025**	**68.75**	**0.01**	**3.00**	**11.42**	**< 0.01**		**1.19**	
Turdus merula*	Partial	**0.010**	**112.30**	**< 0.01**	**2.82**	**5.643**	**< 0.05**		**0.38**	
Turdus philomelos*	Partial	**0.054**	**9.63**	**0.07**	**2.64**	**40.02**	**< 0.01**		**2.62**	
***Troglodytes troglodytes***	Partial	0.009	30.31	< 0.01	1.85	3.37	0.07	48.21	1.05	

* Species where regressions were conducted using robust standard errors due to heteroscedasticity

All handling records were requested from the BTO for 3 main bird observatories: Gibraltar Point (53.10°N, 0.33°W), Rye Bay (50.90° N, 0.76°W) and Portland Bill (50.51°N, 2.45°W), chosen because they are on the east, south and south coasts of the UK respectively, so likely represent records at final points of land contact before the birds left the UK and began their migration (see below for analysis of *M*_b_ distributions across the sites, which supports this statement).

### Sample sizes and data cleaning

To examine departure *M*_f_ for autumn migration only, the original data set was reduced from 418,175 records by including only adults (Euring age code 4 and above) caught between July and October. This period of 4 months was chosen to accommodate the possibility of changing phenology over the 56 years. The assumption was that birds captured in this period were either on migration or finished/nearly finished breeding, while we avoided including winter arivals of some species e.g. *Sturnus vulgaris*. The Tidyverse package in R [34] was used to remove duplicate individuals by ring number (20,440 records remaining). Outliers, assumed to be errors in original measurements or data entry were removed (Supplementary table i). Records were also deleted due to missing data for some measurements, and when *l*_wing_ and *M*_b_ were combined in *M*_f_ (%) and *M*_b-lean_ (lean body mass, kg) calculations, further decreases in sample size occurred due to missingness of one or both variables (Supplementary table i).

### Morphological changes

**Fig. 1:**
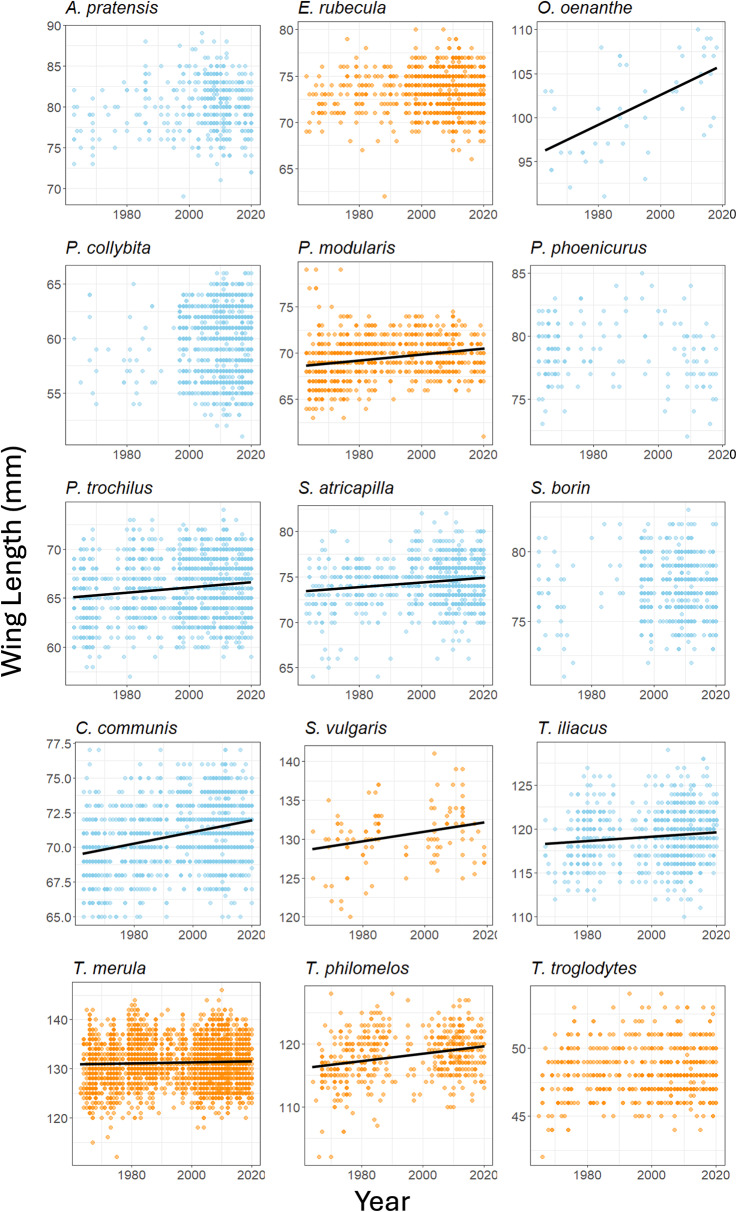
Changes in wing length between 1964 and 2020 for each of the 15 species. Nine species showed an increase in wing length (lines of best fit are included for these species) whilst there was no detectable change in 6 species (see Table 1 for lines of best fit and regression analyses output). Partial migrants are shown with blue dots and full migrants with orange

**Fig. 2:**
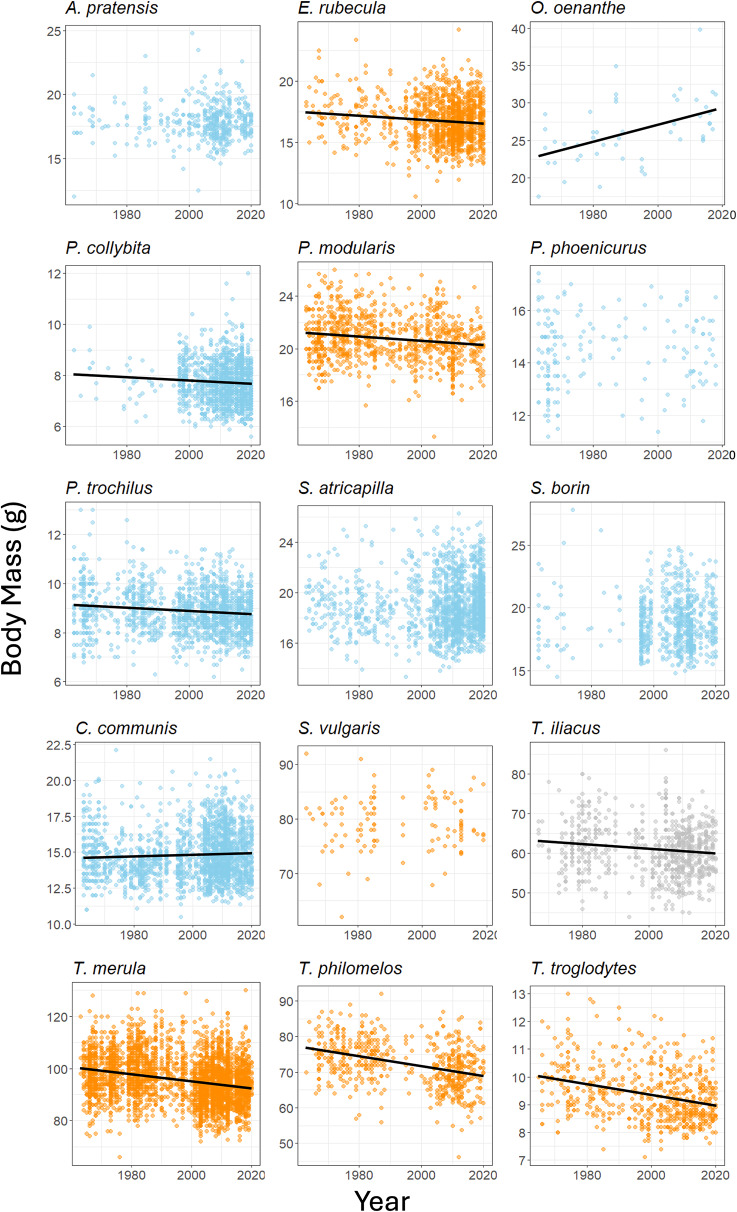
Changes in body mass between 1964 and 2020 for each of the 15 species. Eight species experienced a decrease in body mass, 2 an increase (lines of best fit are included for these species) and for 5 there was no detectable change (see Table 2 for lines of best fit and regression analyses output). Partial migrants are shown with blue dots and full migrants with orange. *T. iliacus* is shown with grey dots as it is a winter migrant so was not included in comparisons of body mass between migratory strategies

Linear regressions were used to ascertain whether *l*_wing_ (Fig. 1), *M*_b _ (Fig. 2), *M*_b−lean_, and *M*_f_ changed predictably between 1964 and 2020. For those that showed a change, the formula of the regression line (*y = mx + b*) gave estimates of *l*_wing_, *M*_b_, *M*_b−lean_ and *M*_f_ for 1964 and 2020, which were used in subsequent flight range calculations. In species which did not change significantly, the mean value across all years was used in the 1964 and 2020 calculations. Some records go back to 1960 or 1963 (Supplementary table i) and these years were used in generation of the predictive linear models, but the years compared were 1964 and 2020 for all species.

**Table 2:** The results of linear regression of body mass (*M*_b_) against year by species, including changes between 1964 and 2020 and mean *M*_*b*_ for those which showed no significant change

Species	Gradient (*m*)	Intercept (*b*)	Adjusted *R*²	Standard error of residuals	*F*	*P*	Mean *M*_b_ (g)	Δ *M*_b_ (%)
***A. pratensis***	< 0.001	18.15	0.00	1.39	0.00	0.99	17.87	-0.06
***E. rubecula***	**-0.016**	**49.19**	**< 0.01**	**1.69**	**15.75**	**< 0.01**		**-5.20**
***O. oenanthe***	**0.113**	**-198.17**	**0.21**	**3.77**	**13.98**	**< 0.01**		**27.38**
***P. collybita***	**-0.007**	**20.80**	**0.00**	**0.78**	**6.64**	**0.01**		**-4.53**
***P. modularis***	**-0.017**	**54.16**	**0.03**	**1.72**	**26.1**	**< 0.01**		**-4.43**
***P. phoenicurus***	0.005	4.57	< 0.01	1.37	0.83	0.36	14.36	1.94
P. trochilus*	**-0.007**	**23.04**	**0.02**	**1.288**	**25.32**	**< 0.01**		**-4.34**
S. atricapilla*	0.004	11.90	< 0.01	2.01	0.79	0.37	18.95	1.05
S. borin*	0.002	14.92	< 0.01	1.94	0.08	0.7788	18.84	0.58
C. communis*	**0.009**	**-2.62**	**< 0.01**	**1.79**	**13.41**	**< 0.01**		**3.37**
***S. vulgaris***	0.009	61.41	< 0.01	4.19	0.11	0.75	79.42	0.64
T. iliacus*	**-0.060**	**180.34**	**0.02**	**3.41**	**14.16**	**< 0.01**		**-5.28**
***T. merula***	**-0.136**	**368.08**	**0.08**	**8.07**	**312.30**	**< 0.01**		**-7.64**
***T. philomelos***	**-0.140**	**352.11**	**0.15**	**5.89**	**100.50**	**< 0.01**		**-10.24**
T. troglodytes*	**-0.020**	**48.89**	**0.10**	**1.323**	**66.74**	**< 0.01**		**-11.04**

* Species where regressions were conducted using robust standard errors due to heteroscedasticity

**Fig. 3:**
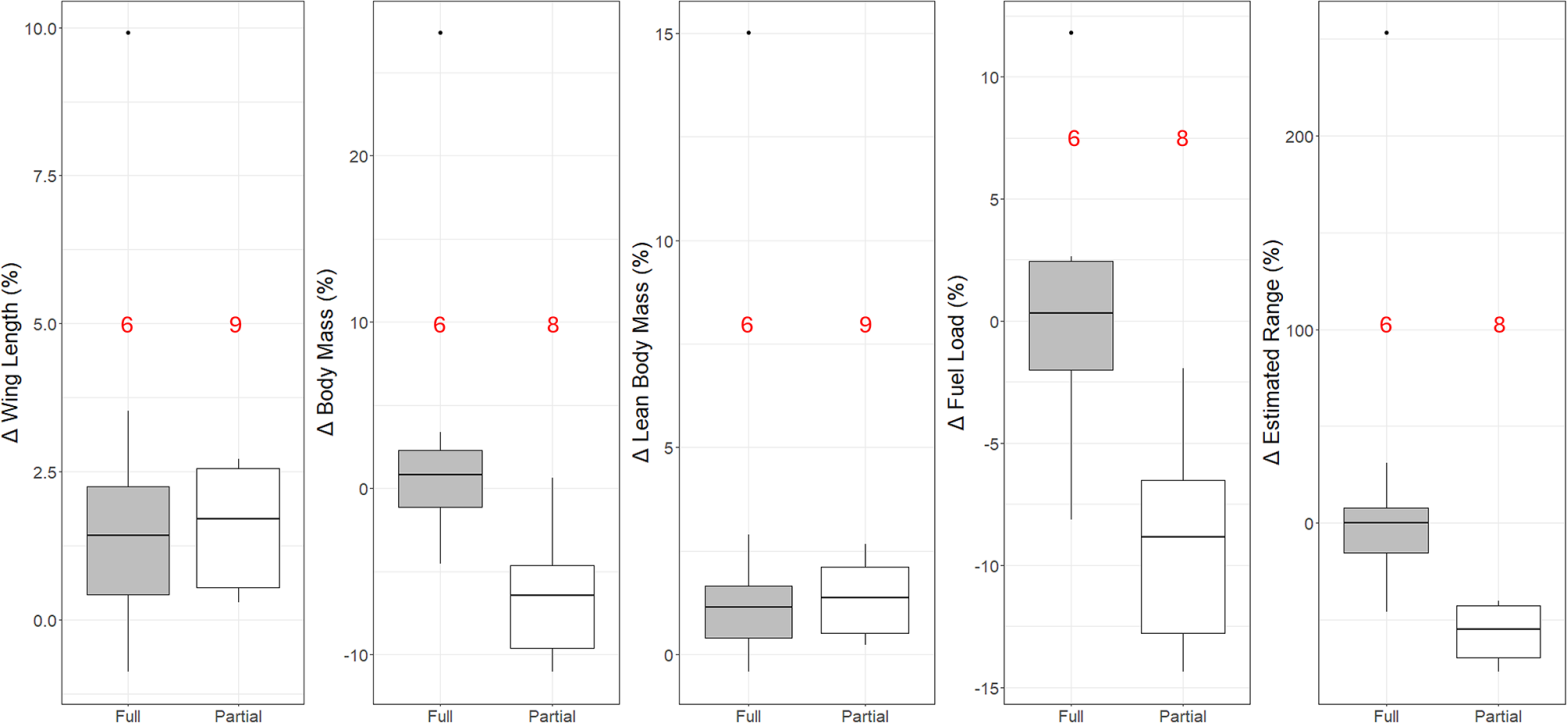
Changes in morphology in partial and full migrants between 1964 and 2020. Wing length (*U* = 25, *P* = 0.86) and lean body mass (*U* = 0.29, *P* = 0.86) did not differ between migratory strategies. In contrast, a decrease in body mass was evident in partial migrants and this differed from the no-change observed for full migrants (*U* = 5, *P* = 0.01). Partial migrants showed a greater decrease in fat mass than did full migrants (*U* = 2.5, *P* < 0.01). Estimated changes in flight range also differed (*U* = 3, *P* < 0.01) between full and partial migrants with the former estimated to have experienced no change, whilst partial migrants experienced a decrease in range. Number of species are given in red above the bars, and dots represent outlying species. Medians and quartiles (Q1, Q3) are: **Δ Wing Length (%): Full** = 1.43 (0.43, 2.25), **Partial** = 1.70 (0.55, 2.55). **Δ Body Mass (%): Full** = 0.82 (-1.13, 2.30), **Partial** = -6.42 (-9.59, -4.62). **Δ Lean Body Mass (%): Full** = 1.14 (0.41,1.65), **Partial** =1.38 (0.52, 2.11). **Δ Fuel Load (%): Full** = 0.33 (-2.02, 2.46), **Partial** = -8.85 (-12.78, -6.52). **Δ Estimated Range (%): Full** = 0.00 (-15.18, 7.80), **Partial** = -54.60 (-69.53, -42.38)

In terms of migratory strategy, there were no detectable differences in *l*_wing_ or *M*_b−lean_ between partial and full migrants, but changes in *M*_b_ and *M*_f_ did differ between birds of different migratory strategies, with partial migrants experiencing a greater median decrease in both parameters of mass (Fig. 3).

Nine out of the 15 species analysed for changes between 1964 and 2020 showed a decrease in the estimated non-stop flight range of between 20.7% and 70.2*%*, whilst 3 species did not show a change in estimated flight range (Table 5). Partial migrants had a larger median decrease in range than full migrants (Fig. 3).

### Estimating fuel load

*M*_b−lean_ was estimated from Kelsey et al. [32] as:

1 $${M_{b - lean}} = {\beta _0} + {\text{ }}{\beta _2} \times {l_{wing}}$$

and the coefficients *β*_0_ and *β*_2_ were taken from Table 2 of Kelsey et al. [32]. *M*_f_ (%) was calculated as:

2 $${M_f} = (({M_b} - {M_{b - lean}})/{M_{b - lean}}) \times 100$$

This relationship between *M*_b-lean_ and *l*_wing_ was derived from birds captured on Helgoland, an island situated approximately 50 km from the German coastline. Both full and partial migrants are routinely captured there during migration. Birds passing across the island are crossing the German Bight so have not undergone long over-sea migrations. Instead, their migrations would largely be across “common landscape” enabling refuelling on route to maintain fat stores and, hence, removing the need to use lean mass protein as a fuel source [35]. Furthermore, it is thought that the organs of the body in passerines (for example, the small intestine) are used before flight muscle if lean mass needs to be catabolized during periods of nutrient scarcity [36]. Therefore, the relationship between between *M*_b-lean_ and *l*_wing_ described in Eq. 1 should be appropriate to the birds included in this current study.

### Estimating ranges

FlyingR [37] was used to estimate flight ranges (the maximum non-stop, where no feeding occurs, distance achievable from departing the UK) for each species for 1964 and 2020, which requires the following morphological parameters: mass (*M*_b_, kg), wingspan (*b*, m), mass of fat (*M*_fat_, kg) and muscle mass (*M*_m_, kg). Estimates of *M*_b_ were derived for 1964 and 2020 from the linear regressions (Fig. 2 and Table 2). The BTO data only provided *l*_wing_, so values for *b* were obtained from Cramp et al. (1977-94) [38]. These were validated because they were paired with values for *M*_b_ by Bruderer and Boldt [35] that were close to those in the BTO data. Similarly, wing areas (*A*) were originally taken from the Meinertzhagen and Basel Museum (unpubl. data), from Bruderer and Boldt [39]. Flight range calculations are extremely sensitive to changes in wingspan (*b)* [40], so, for each species, these were calibrated to changes in *l*_wing_ derived from the linear regressions. Values from Cramp et al. were taken as *b* for 1964 and increased by the same percentage as *l*_wing_ for 2020.

3 $${M_{f{\text{ }} = }}{M_b}-{M_{b - lean}}$$

Where *M*_b_ is calculated from the regression equations derived from the BTO data and *M*_b−lean_ calculated from Eq. 1. *M*_m_ was calculated as 21% of *M*_b−lean_ [41]. Air density, also required to calculate flight range, was set to the value at 500 m above sea level, 1.17 kg/m^3^ [30]. The change in range (%) per species was calculated as:

4 $$\begin{gathered}\Delta range{\text{ }}\left( \% \right) = \hfill \\\,\,\,\,\,\,\,\,\,\,\,\,\,\,\,\,\,\,\,\,\,\,\,\,\,\,\,\,\left( {\left( {range{\text{ }}in{\text{ }}2020 - range{\text{ }}in{\text{ }}1964} \right)/range{\text{ }}in  1964} \right) \times 100 \hfill \\ \end{gathered}$$

### Statistical analyses

R v4.0.2 [42] was used for all statistical analyses and *P* values less than 0.05 considered as statistically significant. In all cases adjusted *R*^2^ values are reported. For all but two species, the data approximated to a normal distribution. The *l*_wing_ data for *Phylloscopus collybita*, and *Phylloscopus trochilus*, however, showed a double Gaussian distribution. Nonetheless, the data distribution was so symmetrical that the mean and median were equal, and regression analysis is reasonably robust to non-normal data distributions. Consequently, Gaussian linear regression was used for these as well. Heteroscedasticity was identified within the data included in 24 of the regressions (Marked with (*) in Tables 1, 2, 3 and 4). For these 24 regression analyses robust standard errors were used. Two-way ANOVAs with an interaction term were used to test for possible differences in *M*_b_ and *l*_wing_ among the 3 bird observatories, whilst controlling for species differences.

**Table 3:** The results of linear regression of lean body mass (*M*_*b-lean*_, Eq. 1) against year by species, including changes (∆) between 1964 and 2020 and mean *M*_*b-lean*_ for those which showed no significant change

Species	Gradient (*m*)	Intercept (*b*)	Adjusted *R*²	Standard error of residuals	*F*	*P*	Mean *M*_b−lean_ (g)	Δ *M*_b−lean_ (%)
***A. pratensis***	0.004	6.42	0.00	0.70	3.04	0.82	15.38	1.65
***E. rubecula***	0.001	13.60	< 0.01	0.34	0.58	0.45	14.88	0.24
***O. oenanthe***	**0.058**	**-92.27**	**0.32**	**1.48**	**22.94**	**< 0.01**		**15.01**
***P. collybita***	0.001	5.82	< 0.01	0.33	0.22	0.64	6.83	0.41
P. modularis*	0.007	3.78	0.05	1.06	42.21	**< 0.01**		2.25
***P. phoenicurus***	-0.001	15.07	0.01	0.19	1.69	0.20	13.10	-0.42
***P. trochilus***	**0.004**	**-0.19**	**0.02**	**0.45**	**34.14**	**< 0.01**		**2.89**
S. atricapilla*	**0.002**	**11.90**	**0.02**	**0.64**	**28.17**	**< 0.01**		**0.79**
S. borin*	< 0.001	16.20	< 0.01	0.37	0.35	0.55	16.53	0.12
C. communis*	**0.003**	**7.68**	**0.09**	**0.50**	**189.3**	**< 0.01**		**1.14**
***S. vulgaris***	**0.036**	**4.77**	**0.06**	**2.10**	**8.61**	**< 0.01**		**2.67**
***T. iliacus***	**0.016**	**23.57**	**0.01**	**1.92**	**11.42**	**< 0.01**		**1.63**
T. merula*	**0.005**	**77.79**	**< 0.01**	**2.13**	**5.64**	**< 0.05**		**0.34**
T. philomelos*	**0.019**	**25.20**	**0.07**	**1.54**	**40.02**	**< 0.01**		**1.68**
***T. troglodytes***	0.002	5.29	< 0.01	0.33	3.37	0.07	8.51	1.07

* Species where regressions were conducted using robust standard errors due to heteroscedasticity

**Table 4:** The results of linear regression of fuel load (*M*_f_, Eq. 2) against year by species, including change (∆) between 1964 and 2020 and mean *M*_f_ for those which showed no significant change

Species	Gradient (*m*)	Intercept (*b*)	Adjusted *R*²	Standard error of residuals	*F*	*P*	Mean *M*_f_ (%)	Δ *M*_f_ (%)
***A. pratensis***	-0.028	72.94	< 0.01	8.88	0.75	0.39	16.30	-0.67
E. rubecula*	-0.104	221.20	0.01	4.45	14.04	< 0.01		-5.83
***O. oenanthe***	0.211	-407.76	0.05	14.11	3.34	0.07	12.33	11.80
***P. collybita***	**-0.108**	**230.94**	**< 0.01**	**9.35**	**12.10**	**< 0.01**		**-6.06**
***P. modularis***	**-0.153**	**318.73**	**0.07**	**9.38**	**67.26**	**< 0.01**		**-8.57**
***P. phoenicurus***	0.047	-84.16	< 0.01	10.51	1.28	0.26	9.65	2.65
P. trochilus*	**-0.081**	**135.81**	**0.04**	**3.56**	**63.31**	**< 0.01**		**-8.12**
S. atricapilla*	0.001	12.01	< 0.01	4.87	0.00	0.95	14.79	0.08
S. borin*	0.010	-6.68	< 0.01	4.77	0.06	0.82	13.95	0.58
***C. communis***	**0.043**	**-70.63**	**< 0.01**	**0.14**	**5.49**	**< 0.05**		**2.40**
***S. vulgaris***	-0.035	74.62	< 0.01	6.51	0.82	0.37	5.19	-1.95
T. iliacus*	**-0.140**	**289.47**	**0.02872**	**4.74**	**24.21**	**< 0.01**		**-7.84**
***T. merula***	**-0.163**	**332.99**	**0.09**	**8.60**	**381.60**	**< 0.01**		**-9.12**
***T. philomelos***	**-0.257**	**528.30**	**0.19**	**9.29**	**128.10**	**< 0.01**		**-14.37**
T. troglodytes*	**-0.250**	**509.40**	**0.1438**	**4.14**	**99.23**	**< 0.01**		**-14.00**

* Species where regressions were conducted using robust standard errors due to heteroscedasticity

**Table 5:** The calculated raw flight range estimates from FlyingR (estimated range), estimated changes (∆) in flight range for each species (eq. 4), and contributing variables as follows: wingspan *b*(m), mass of fat *M*_fat_ (kg), wing area *A*_wing_ (m^2^), and muscle mass *M*_m_ (kg)

	1964					2020					
Species	*b *(m)	*M*_fat_ (kg)	*A*_wing_ (m^2^)	*M*_m_ (kg)	Estimated Range (km)	*b* (m)	*M*_fat_ (kg)	*A*_wing_ (m^2^)	*M*_m_ (kg)	Estimated Range (km)	Δ Estimated Range (%)
***A. pratensis***	0.235	0.0025	0.011	0.003	1132	0.235	0.0025	0.011	0.003	1132	0.0
***E. rubecula***	0.210	0.0025	0.010	0.003	1057	0.210	0.0016	0.010	0.003	631	-40.3
***O. oenanthe***	0.290	0.0014	0.014	0.005	479	0.319	0.0045	0.014	0.005	1691	253.0
***P. collybita***	0.180	0.0012	0.007	0.001	1048	0.180	0.0008	0.007	0.001	654	-37.6
***P. modularis***	0.200	0.0032	0.009	0.004	1095	0.205	0.0019	0.009	0.004	605	-44.7
***P. phoenicurus***	0.223	0.0013	0.010	0.003	624	0.223	0.0013	0.010	0.003	624	0.0
***P. trochilus***	0.193	0.0015	0.007	0.002	1308	0.197	0.0009	0.007	0.002	708	-45.8
***S. atricapilla***	0.215	0.0026	0.009	0.003	1059	0.219	0.0024	0.009	0.003	977	-7.7
***S. borin***	0.223	0.0023	0.009	0.003	955	0.223	0.0023	0.009	0.003	955	0.0
***C. communis***	0.208	0.0017	0.007	0.003	909	0.215	0.0021	0.007	0.003	1192	31.2
***S. vulgaris***	0.395	0.0049	0.022	0.016	578	0.406	0.0029	0.022	0.016	338	-41.6
***T. iliacus***	0.340	0.0080	0.023	0.012	1127	0.344	0.0038	0.023	0.012	490	-56.5
***T. merula***	0.363	0.0116	0.028	0.019	948	0.364	0.0037	0.028	0.019	273	-71.2
***T. philomelos***	0.345	0.0151	0.021	0.013	2291	0.354	0.0063	0.021	0.013	814	-64.5
***T. troglodytes***	0.150	0.0015	0.005	0.002	966	0.150	0.0004	0.005	0.002	224	-76.8

Comparisons (1964 v 2020) of changes in *l*_wing_, *M*_b_, *M*_b−lean_, *M*_f_, estimated range among migratory strategy were made using Mann-Whitney *U*-tests, as sample sizes were small with a single value for each variable per species, resulting in non-normal data distributions. *P*-values are not adjusted (e.g., Bonferroni correction) because such protocols are particularly harsh on small sample sizes [43]. Bonferroni corrections, if applied here, however, would not negate any of the reported statistically significant effects. The species were divided into partial and full migrants, with *T. iliacus* excluded from mass, fuel load, and flight range comparisons due to its unique migratory strategy (refer to Methods: study sites and species).

## Results

As would be expected, the majority of the variation in *M*_b_ was accounted for by species (*F*_14, 15981_ = 70059.53, *r*^2^ = 0.91, *P* < 0.001) and, although there was an overall difference in *M*_b_ among the 3 observatories (*F*_2, 15981_ = 41048.62, *r*^2^ = 0.07, *P* < 0.001), it was driven by the interaction effect: the pattern in *M*_b_ variation among the 3 observatories was inconsistent among species (observatory x species: *F*_28, 15981_ = 64.73, *r*^2^ < 0.01, *P* < 0.001). There is no systematic effect of observatory on *M*_b_, with six species heaviest at Gibraltar point (*Turdus merula*, *Prunella modularis*,* Sylvia borin*, *T. iliacus*, *Turdus philomelos* and *Troglodytes troglodytes*), six at Portland bill (*Sylvia atricapilla*, *Atricapilla pratensis*, *Erithacus rubecula*, *S. vulgaris*, *Oenanthe oenanthe* and *P. trochilus*) and three at Rye bay (*P. collybita*, *Phoenicurus phoenicurus* and *Curruca communis*). The results for *l*_wing_ were similar. The anticipated difference in *l*_wing_ among species was evident (*F*_14, 15860_ = 94466.23, *r*^2^ = 0.92, *P* < 0.001) and, again, although there was an overall difference among observatories (*F*_2, 15860_ = 50813.90, *r*^2^ = 0.07, *P* < 0.001), it was once more driven by the interaction between observatory and species (*F*_28, 15860_ = 11.93, *r*^2^ < 0.01, *P* < 0.001). Therefore, *l*_wing_ was also not affected systematically by observatory location. Nine species had longest recorded *l*_wing_ at Rye bay (*P. modularis*,* S. borin*, *T. iliacus*, *T. philomelos*, *T. troglodytes*, *A. pratensis*, *S. vulgaris*, *P. trochilus* and *C. communis*), four at Portland bill (*S. atricapilla*,* P. collybita*, *E. rubecula* and *O. oenanthe*) and two at Gilbraltar point (*T. merula* and *P. phoenicurus*).

Nine of the 15 species showed increases in *l*_wing_, (0.4 − 9.9%) and 6 showed no detectable changes in *l*_wing_ (Fig. 1 and Table 1). Eight species exhibited a decrease (-4.4 to -11.0%) in *M*_b_ between 1964 and 2020 (Fig. 2 and Table 2), 5 showed no change and, contrastingly, *O. oenanthe* and *C. communis* increased their *M*_b_ (Table 2). Nine species showed an increase in *M*_b−lean_ (0.3 − 15.0%), while six showed no change (Table 3). The *M*_f_ of 8 species’ decreased (-6.1 and − 14.4%), one species, *C. communis*, had increased *M*_f_, and 6 species showed no change in *M*_f_ (Table 4).

## Discussion

The hypothesis that migrant passerines will have undergone morphological changes over the course of 56 years was supported by our results. 12 of the 15 species showed morphological changes between 1964 and 2020 in at least one of the 4 variables measured. Furthermore, accounting for these morphological changes in predictive algorithms [32] and flight models [40] indicates that migratory fuel load and estimated non-stop flight ranges may have also changed. The fact that, although most flight ranges were estimated to decrease, some did not change, while others increased, indicates that different species have reacted to a period of climate change differently.

Previous studies have shown that linking thermoregulatory requirements to long-term changes in morphology should be done cautiously [23], as there may be non-stationarity of effects across species of differing body sizes [19]. In addition, other abiotic factors can have similar effects, for example, elevation [44, 45], and the effects of Bergmann’s rule which could be mitigated by changing phenology [23]. The morphological change documented here, coincides with a period during which climate change has occurred. What, however, has specifically driven the changes seen during this period (1964–2020) is not clear and is not the focus of our study. Irrespective of whether the morphological changes found are driven by thermoregulatory requirements or alternative factors, such as changing geographical ranges or breeding phenology, they coincide with a period of climate change, and must affect flight performance.

The inconsistent effect of year on body mass (*M*_b_) with decreases in eight species, no significant change in five species and increases in two species, mirrored the level of variation among species found in previous work [20, 21]. It is likely, however, that inconsistences in the effects of year upon *M*_b_ are both species-specific, and study specific. For example, Yom-Tov et al. [20] found an increase in *T. merula M*_b_, whereas, here, we found a decrease. Their [20] study site was a single inland woodland, so could have contained a larger proportion of sedentary individuals, thus accounting for some differences in results. Additionally, in our study, large sections of the south and east coasts were sampled including sub-optimal habitats, such as farmland: *T.merula* do better in woodland [46].

Most species showed between − 2 and + 2% (-0.056 and + 0.056% year ^− 1^) change in wing length (*l*_wing_) over the study period. The increases are of a similar magnitude to the findings of previous studies, where long-term increases in passerine *l*_wing_ were also reported in California [21] as well as in other studies based on BTO data [20]. The increases in *l*_wing_ found previously in England, however, were non-linear in *T. troglodytes*,* P. modularis* and *T. merula* [20]. Non-linear changes are an aspect which could be explored further but here, for simplicity, structural change was modelled as a linear relationship. It is known that wing length in birds is often dependent on migratory strategy, especially where other lifestyle constraints such as breeding habitat do not compete [15, 47]. For example, the *l*_wing_ of *Oenanthe* species is affected by migratory strategy, with medium-sized wings in partially migrant species (e.g. *O. lugens*), long wings in long-distance migrants (*O. oenanthe* and *O. isabellina*), and short rounded wings in sedentary species (e.g. *O. pileata*) [47]. *O. oenanthe* is also thought to fuel before departure in proportion to the distance to the next stopover [46] and this, and several other species, appear to have made a westward geographical range shift in the north [48], resulting in an increase in required flight range to reach wintering grounds in sub-saharan Africa. Apparent increases in required migratory distance and fuelling that we see in *O. oenanthe* (estimated here as 186%), linked to an increase in *l*_wing_ of 9.91%, are in accordance with increased flight range requirements.

Overall, changes in *l*_wing_ and lean body mass (*M*_b−lean_) did not differ between birds of different migratory strategy. The increases in *l*_wing_ and *M*_b−lean_ observed here for some species (Tables 1 and 3), however, constitutes a structural increase in body size. If these changes are not proportional, wing loading will be affected, in turn influencing the flight speed corresponding to the most energy efficient velocity. For example, lower wing loading reduces the speed corresponding to the most energy efficient flight and consumes less energy overall, potentially increasing non-stop flight range [49]. The mean *M*_f_ of most species decreased or did not change (Table 4) despite interspecific difference in changes of *l*_wing_, *M*_b_ and *M*_b−lean_. It could be that *M*_f_ decreases are driven by changes in available nutrition prior to departure. All other morphological parameters being equal, a reduced *M*_f_ will reduce non-stop flight ranges, and it is possible that the changes in *l*_wing_, *M*_b_ and *M*_b−lean_ are a compensation for a reduced *M*_f_ to allow species to continue to fly the same distances. It is pertinent to note that different combinations of *l*_wing_, *M*_f_ and *M*_b−lean_ can result in the same estimated non-stop flight range and, therefore, different compensatory strategies can achieve the same result when focusing on flight range only. Like the results reported here, a reduced body size coupled with increased *l*_wing_ was found across 52 North American passerine species [18], and suggested to be a thermoregulatory body size decrease concomitant with climate change, coupled with an increased *l*_wing_ to compensate and maintain migration distances [18].

Those species which show decreases in estimated flight ranges (*P. trochillus*,* P. collybita*, and *T. illiacus*) may not, due to life-history constraints, be compensating sufficiently, and therefore will require their first stopover sooner and perhaps, more stopovers to reach their migration destination [30]. This highlights the importance of migratory stopovers, for example, those in the Mediterranean region and North Africa, with their use potentially increasing and, consequently, their conservation becoming more vital. In contrast, those species with little change or increases in estimated range may be compensating successfully with structural changes to make up for reduced *M*_f_. Changes in morphology, migratory behaviour and fuelling strategies are not mutually exclusive, which might explain the variation in range changes and biometrics among species.

The estimated increase in non-stop flight range on departure from the UK since 1964, apparent in some species (all of which are long-distance migrants) supports the theory that flight range requirements are increasing, as stopover opportunities before and after geographical barriers may be becoming less predictable and migrants need to cross larger areas of inhospitable terrain [50–52]. The decrease in estimated flight ranges for partial migrants was greater than that of full migrants. Partial migrants possess more migratory behavioural plasticity when making decisions based on environmental cues and fuel loading [53]. Environmental changes may mean that partial migrants adapt behaviourally, migrating shorter distances or becoming permanently resident species. A potential benefit of not needing to migrate is removing the trade-off between optimum morphology for their breeding habitat and migratory flight. There is potentially greater selection pressure on obligate migratory species to adapt morphologically to climate change as they have less opportunity to alter migratory strategy [54, 55], i.e., destination and stopover sites. Nevertheless, some full migrants do show a degree of plasticity and are able to change strategy, for example, an increase in proportion of short-winged short distance migrants compared to long-winged long-distance populations of *S. atricapilla* has been recorded migrating through the southern Baltic [56].

In the short term, it may be that instead of seeing migratory strategy changes, we will see higher mortality rates in those species that are unable to adapt quickly [57]. Mortality rates on migration are difficult to quantify but it is known that variation in annual survival relies heavily on survival during migration [58–60]. Population declines in British obligatory migrants have been recorded since the 1970s, linked to droughts in the Sahel and temporal mismatches in food requirement and availability - key impacts of climate change [61, 62]. We would perhaps expect to see a shift to a greater proportion of the population not migrating (and surviving) in partial migrants and higher mortality on migration in full migrants in the short term, until they can adapt sufficiently. This change in composition of the population would occur whether individuals are facultative migrants who ‘choose’ to change strategy, or whether there is evolutionary change throughout a population towards a higher proportion of sedentary individuals.

Our narrative has very much focused on the idea that it is the need to maintain flight range that drives morphological changes. This is not an unrealistic supposition, particularly if changes to extrinsic factors occur gradually. It is, however, pertinent to acknowledge that species may adapt to changing extrinsic conditions initially by changing their behaviour (e.g. stopping off more frequently during migration) and any changes in morphology may be driven by the requirements of the new behavioural strategy. Such changes in strategy, though, may be less likely as anthropogenic activities lead to the increasing fragmentation of the high-quality habitat necessary to facilitate successful stopovers.

It is important to acknowledge the limitations of our study, particularly with respect to Eqs. 1 and 2 [32], with the former for each of the species having adjusted *R*^2^ values of ≤ 0.52. To date, Eqs. 1 and 2 provide the only options for predicting passerine fuel loads based on commonly taken measurements, across large historic samples no longer available for invasive manipulation. However, they ultimately lead to results with very broad error bands when applied to larger samples than they were derived from. Here, in all species the beginning (1964) and end (2020) of the fitted regression line remained above zero despite some individual points falling below (negative fuel loads). Species were compared based on change in *M*_f_, and average *M*_fat_ remained positive. Furthermore, some validation of *M*_f_ estimates is possible, as our estimates are comparable to *M*_f_ found in mid-migration passerines. For example, the fuel load of *T. troglodytes* at Baltic stopovers was 5.3% between 1994 and 2006, while, here, the departure *M*_f_ estimation for *T. troglodytes* in 2020 was 4.4% [63]. The *M*_f_ of migrants at stopovers in Morocco also had similar values to our estimates. *P. phoenicurus* had *M*_f_ of 0.12 at two out of three of the study sites, compared to our estimate of 0.09 [63]. Similarly, *P. trochilus* were recorded as having a mean *M*_f_ of 0.16 at two sites and 0.06 at a third (all +/- approx. 0.02), while we estimated that *P. trochilus* in 2020 had a mean departure *M*_f_ of 0.12 [64].

Several assumptions were necessary in this study. The use of pre-existing databases for wingspan (*b*) and wing area (*A*) were required due to the lack of these in the BTO data set. *b* is highly influential on flight speed and power calculations, but the assumption that changes in *b* and *A* are proportional to changes in *l*_wing_ is reasonable. Generalisations in *M*_b_ and *M*_f_ were minimised by choosing only birds with EURING age code 4 and above measured between July and October with the aim of ensuring only departure fuel loads of adults were estimated. Previously a 95% fuel depletion was used as the threshold for refuelling [30], whereas, here, the flight range was assumed to be the distance an individual could travel from the UK before the fuel load was fully depleted and the bird had to make the first stopover. Finally, we used mean values for all variables required to estimate *M*_f_, which could result in the estimates comprising birds at all stages of the fattening process, potentially leading to an underestimate of non-stop flight range. However, the same approach was used for all annual means throughout the paper meaning that the changes between 1964 and 2020 are robust, even if the *M*_f_ values trend towards underestimates. Furthermore, using maximum values for all variables could lead to mis-leading results due to the existence of outlying data points (some of which could be measurement error) and the inherent propagation of errors associated with estimating *M*_f_. Calculating flight ranges provides some degree of interpretability - even if it is not perfect.

It can be concluded that several British passerines have undergone morphological changes and decreases in estimated fuel load between 1964 and 2020. These changes differ among species, as well as among studies, highlighting that morphological change and the impacts of climate change are unlikely to have a universal predictable effect. Further studies of site and species-specific conditions across a larger sample of migratory strategies are needed to identify the causes of this variation. Decreases in body mass, fuel load, and consequently flight range are greatest in partial migrants. The overall implications of these results are that many species are undergoing morphological changes which potentially impact their flight range, and some birds may adapt behaviourally or morphologically better than others. Species with reduced estimated non-stop flight ranges but unchanged migratory requirements will have to stop more often to refuel on migration, highlighting the importance of conserving stop-off sites. Particularly, however, those that occur immediately before major geographic barriers, for example, the Mediterranean region and North Africa that precede long sea or desert crossings.

### Electronic supplementary material

Below is the link to the electronic supplementary material.

Supplementary Material 1: Table_i: contains sample sizes, number of standard deviations to which outliers were removed, and years from which data was included.

## Data Availability

The dataset supporting the conclusions of this article is available in the British Trust for Ornithology repository, at https://www.bto.org/our-science/data/data-request-system.

## References

[1] Summary for Policymakers in Climate Change. 2021: The Physical Science Basis. Contribution of Working Group I to the Sixth Assessment Report of the Intergovernmental Panel on Climate Change; 2021 (eds. Masson-Delmotte, V., https://www.ipcc.ch/report/ar6/wg1/

[2] Wormworth JA, Mallon K. Bird Species and Climate Change: The Global Status Report: A synthesis of current scientific understanding of anthropogenic climate change impacts on global bird species now, and projected future effects; 2006. http://pandora.nla.gov.au/tep/67826

[3] Hüppop O, Hüppop KH. North Atlantic Oscillation and timing of spring migration in birds. Proc Royal Soc Lond Ser B: Biol Sci. 2003;270:233–40. 10.1098/rspb.2002.2236 PMC1691241 12614571

[4] Jenni L, Kéry M. Timing of autumn bird migration under climate change: advances in long–distance migrants, delays in short–distance migrants, Proceedings of the Royal Society of London. Series B: Biological Sciences. 2003;270:1467–1471. 10.1098/rspb.2003.2394 PMC1691393 12965011

[5] Miles WT, Bolton M, Davis P, Dennis R, Broad R, Robertson I, Riddiford NJ, Harvey PV, Riddington R, Shaw DN, Parnaby D, Reid JM. Quantifying full phenological event distributions reveals simultaneous advances, temporal stability and delays in spring and autumn migration timing in long-distance migratory birds. Glob Change Biol. 2017;23:1400–14. 10.1111/gcb.13486 27670638

[6] Pinszke A, Remisiewicz M. Long-term changes in autumn migration timing of garden warblers *Sylvia borin* at the southern baltic coast in response to spring, summer and autumn temperatures. Eur Zoological J. 2023;90:283–95. 10.1080/24750263.2023.2192239

[7] Nilsson ALK, Lindström Å, Jonzén N, Nilsson SG, Karlsson L. The effect of climate change on partial migration - the blue tit paradox. Glob Change Biol. 2006;12:2014–22. 10.1111/j.1365-2486.2006.01237.x

[8] Schaefer HC, Jetz W, Böhning-Gaese K. Impact of climate change on migratory birds: community reassembly versus adaptation. Global Ecol Biogeogr. 2008;17:38–49. 10.1111/j.1466-8238.2007.00341.x

[9] Hickling R, Roy DB, Hill JK, Fox R, Thomas CD. The distributions of a wide range of taxonomic groups are expanding polewards. Glob Change Biol. 2006;12:450–5. 10.1111/j.1365-2486.2006.01116.x

[10] Barbet-Massin M, Walther BA, Thuiller W, Rahbek C, Jiguet F. Potential impacts of climate change on the winter distribution of afro-palaearctic migrant passerine. Biol Lett. 2009;5:248–51. 19324660 10.1098/rsbl.2008.0715 PMC2665829

[11] Coristine LE, Kerr JT. Temperature-related geographical shifts among passerines: contrasting processes along poleward and equatorward range margins. Ecol Evol. 2015;5:5162–76. 30151121 10.1002/ece3.1683 PMC6102530

[12] Barbet-Massin M, Thuiller W, Jiguet F. The fate of European breeding birds under climate, land‐use and dispersal scenarios. Glob Change Biol. 2012;18:881–90. 10.1111/j.1365-2486.2011.02552.x

[13] Tellería JL, Fernández-López J, Fandos G. Effect of Climate change on Mediterranean winter ranges of two migratory passerines. PLoS ONE. 2016;11:e0146958. 26761791 10.1371/journal.pone.0146958 PMC4711986

[14] Vágási CI, Pap PL, Vincze O, Osváth G, Erritzøe J. Møller AP. Adaptations to migration in birds. Evol Biol. 2016;43:48–59. 10.1007/s11692-015-9349-0

[15] Hedenström A. Adaptations to migration in birds: behavioural strategies, morphology and scaling effects. Philosophical Trans Royal Soc B: Biol Sci. 2008;363:287–99. 10.1098/rstb.2007.2140 PMC2606751 17638691

[16] Perez-Tris J, Telleria JL. Age-related variation in wing shape of migratory and sedentary blackcaps *Sylvia atricapilla*. J Avian Biol. 2001;32:207–13. 10.1111/j.0908-8857.2001.320301.x

[17] Förschler MI, Bairlein F. Morphological shifts of the external flight apparatus across the range of a passerine (Northern Wheatear) with diverging migratory behaviour. PLoS ONE. 2011;6:18732. 10.1371/journal.pone.0018732 PMC3078915 21533160

[18] Weeks BC, Willard DE, Zimova M, Ellis AA, Witynski ML, Hennen M, Winger BM. Shared morphological consequences of global warming in North American migratory birds. Ecol Lett. 2020;23:316–25. 31800170 10.1111/ele.13434

[19] Zimova M, Weeks BC, Willard DE, Giery ST, Jirinec V, Burner RC, Winger BM. Body size predicts the rate of contemporary morphological change in birds. Proc. Natl. Acad. Sci. U.S.A. 2023;120:e2206971120. 10.1073/pnas.2206971120 PMC10193942 37155909

[20] Yom-Tov Y, Yom-Tov S, Wright JJR, Thorne C, Du Feu R. Recent changes in body weight and wing length among some British passerine birds. Oikos. 2006;112:91–101. 10.1111/j.0030-1299.2006.14183.x

[21] Goodman RE, Lebuhn G, Seavy NE, Gardali T, Bluso-Demers JD. Avian body size changes and climate change: warming or increasing variability? Glob Change Biol. 2012;18:63–73. 10.1111/j.1365-2486.2011.02538.x

[22] Bergmann C. Über die Verhältnisse Der Wärmeökonomie Der Thiere zu Ihrer Grö Sse. Gottinger Studien. 1847;3:595–708.

[23] Zimova M, Willard DE, Winger BM, Weeks BC. Widespread shifts in bird migration phenology are decoupled from parallel shifts in morphology. J Anim Ecol. 2021;90:2348–61. 34151433 10.1111/1365-2656.13543

[24] Pilastro A, Spina F. Ecological and morphological correlates of residual fat reserves in passerine migrants at their spring arrival in Southern Europe. J Avian Biol. 1997;28:309. 10.2307/3676944

[25] Klaassen RH, Hake M, Strandberg R, Koks BJ, Trierweiler C, Exo KM, Bairlein F, Alerstam T. When and where does mortality occur in migratory birds? Direct evidence from long-term satellite tracking of raptors. J Anim Ecol. 2014;83:176–84. 24102110 10.1111/1365-2656.12135

[26] Schaub M, Jenni L. Body mass of six long-distance migrant passerine species along the autumn migration route. J für Ornithologie. 2000;141:44–460. 10.1007/BF01651574

[27] Schmaljohann H, Becker PJJ, Karaardic H, Liechti F, Naef-Daenzer B, Grande C. Nocturnal exploratory flights, departure time, and direction in a migratory songbird. J Ornithol. 2011;152:439–52. 10.1007/s10336-010-0604-y

[28] Both C, Van Turnhout CAM, Bijlsma RG, Siepel H, Van Strien AJ, Foppen RPB. Avian population consequences of climate change are most severe for long-distance migrants in seasonal habitats. Proceedings of the Royal Society B: Biological Sciences. 2010;277:1259–1266. 10.1098/rspb.2009.1525 PMC2842804 20018784

[29] Thackeray SJ, Sparks TH, Frederiksen M, Burthe S, Bacon PJ, Bell JR, Botham MS, Brereton TM, Bright PW, Carvalho L, Clutton-Brock TI. Trophic level asynchrony in rates of phenological change for marine, freshwater and terrestrial environments. Glob Change Biol. 2010;16:3304–13. 10.1111/j.1365-2486.2010.02165.x

[30] Howard C, Stephens PA, Tobias JA, Sheard C, Butchart SH, Willis SG. Flight range, fuel load and the impact of climate change on the journeys of migrant birds Proceedings of the Royal Society B: Biological Sciences. 2018;285:2017.2329. 10.1098/rspb.2017.2329 PMC5832701 29467262

[31] Bairlein F. Migratory birds under threat. Science. 2016;354:547–8. 27811252 10.1126/science.aah6647

[32] Kelsey NA, Schmaljohann H, Bairlein F. A handy way to estimate lean body mass and fuel load from wing length: a quantitative approach using magnetic resonance data. Ringing Migration. 2019;34:8–24. 10.1080/03078698.2019.1759909

[33] NOAA National Centers for Environmental information. Climate at a glance: Global Time Series. 2021, https://www.ncdc.noaa.gov/cag/

[34] Wickham H, Averick M, Bryan J, Chang W, McGowan LD, François R, Grolemund G, Hayes A, Henry L, Hester J, Kuhn M. Welcome to the tidyverse. J Open Source Softw. 2019;43:1686. 10.21105/joss.01686

[35] Bauchinger U, Kolb H, Afik D, Pinshow B, Biebach H. Blackcap warblers maintain digestive efficiency by increasing digesta retention time on the first day of migratory stopover. Physiol Biochem Zool. 2009;82:541–8. 19663605 10.1086/603638

[36] Karasov W, Pinchow B, Starck J, Afik D. Anatomical and histological changes in the alimentary tract of migrating blackcaps (Sylvia atricapilla): a comparison among fed, fasted, food-restricted, and refed birds. Physiol Biochem Zool. 2004;77:149–60. 15057725 10.1086/381465

[37] Masinde B, FlyingR. Simulation of bird flight range. R package version 0.2.0.,2021.

[38] Cramp S, Simmons KEL, Perrins CM, editors. The birds of the western palearctic. Oxford: Oxford University Press; 1998.

[39] Bruderer B, Boldt A. Flight characteristics of birds. Ibis. 2001;143:178–204. 10.1111/j.1474-919X.2001.tb04475.x

[40] Pennycuick CJ. Modelling the Flying Bird. Elsevier Academic; 2008.

[41] Rayner JMV. The evolution of vertebrate flight. Biol J Linn Soc. 1988;34:269–87. 10.1111/j.1095-8312.1988.tb01963.x

[42] R Core Team. R: A language and environment for statistical computing. R Foundation for statistical computing, Vienna, Austria;2020. URL https://www.R-project.org/

[43] Nakagawa S. A farewell to Bonferroni: the problems of low statistical power and publication bias. Behav Ecol. 2004;15:1044–5. 10.1093/beheco/arh107

[44] Youngflesh C, Saracco JF, Siegel RB, Tingley MW. Abiotic conditions shape spatial and temporal morphological variation in north American birds. Nat Ecol Evol. 2022;6:1860–70. 36302998 10.1038/s41559-022-01893-x

[45] Sander MM, Chamberlain D. Evidence for intraspecific phenotypic variation in songbirds along elevation gradients in central Europe. Ibis. 2020;162:1355–62. 10.1111/ibi.12843

[46] Hatchwell BJ, Chamberlain DE, Perrins CM. The demography of blackbirds *Turdus Merula* in rural habitats: is farmland a sub-optimal habitat? J Appl Ecol. 1996;33:1114–24. 10.2307/2404691

[47] Kaboli M, Aliabadian M, Guillaumet A, Roselaar CS, Prodon R. Ecomorphology of the wheatears (genus *Oenanthe*). Ibis. 2007;149:792–80. 10.1111/j.1474-919X.2007.00714.x

[48] Virkkala R, Rajasärkkä A. Northward density shift of bird species in boreal protected areas due to climate change. Boreal Environ Res. 2011;16:2–13.

[49] Norberg UM. Vertebrate Flight. Berlin Heidelberg: Springer; 1990.

[50] Bairlein F. Migratory fuelling and global climate change. Birds Clim Change. 2004;35:33–47. 10.1016/S0065-2504(04)35002-6

[51] Potvin DA, Välimäki K, Lehikoinen A. Differences in shifts of wintering and breeding ranges lead to changing migration distances in European birds. J Avian Biol. 2016;47:619–28. 10.1111/jav.00941

[52] Huntley B, Collingham YC, Willis SG, Green RE. Potential impacts of climatic change on European breeding birds. PLoS ONE. 2008;3:1439. 10.1371/journal.pone.0001439 PMC2186378 18197250

[53] Eikenaar C, Ballstaedt E, Hessler S, Klinner T, Müller F, Schmaljohann H. Cues, corticosterone and departure decisions in a partial migrant. Gen Comp Endocrinol. 2018;261:59–66. 29397064 10.1016/j.ygcen.2018.01.023

[54] Packmor F, Klinner T, Woodworth BK, Eikenaar C, Schmaljohann H. Stopover departure decisions in songbirds: do long-distance migrants depart earlier and more independently of weather conditions than medium-distance migrants? Mov Ecol. 2020;8:1–14. 32047634 10.1186/s40462-020-0193-1 PMC7006082

[55] Pulido F, Widmer M. Are long-distance migrants constrained in their evolutionary response to environmental change? Causes of variation in the timing of autumn migration in a blackcap (*S. Atricapilla*) and two garden warbler (*Sylvia borin*) populations. Volume 1046. Annals of the New York Academy of Sciences; 2005. pp. 228–41. 10.1196/annals.1343.020 16055856

[56] Ożarowska A, Zaniewicz G, Meissner W. Blackcaps *Sylvia atricapilla* on migration: a link between long-term population trends and migratory behaviour revealed by the changes in wing length. Acta Ornithologica. 2016;51:211–9. 10.3161/00016454AO2016.51.2.007

[57] Møller AP, Rubolini D, Lehikoinen E. Populations of migratory bird species that did not show a phenological response to climate change are declining. Proceedings of the National Academy of Sciences. 2008;105:16195–16200. 10.1073/pnas.0803825105 PMC2571031 18849475

[58] Robinson RA, Meier CM, Witvliet W, Kéry M, Schaub M. Survival varies seasonally in a migratory bird: linkages between breeding and non-breeding periods. J Anim Ecol. 2020;89:2111–21. 32383289 10.1111/1365-2656.13250

[59] Newton I. Weather-related mass-mortality events in migrants. Ibis. 2007;149:453–67. 10.1111/j.1474-919X.2007.00704.x

[60] Newton I. Migration mortality in birds. Ibis. 2024. 10.1111/ibi.13316. 10.1111/ibi.13316

[61] Winstanley D, Spencer R, Williamson K. Where have all the whitethroats gone? Bird Study. 1974;21:1–14. 10.1080/00063657409476397

[62] Clausen KK, Clausen P. Earlier Arctic springs cause phenological mismatch in long- distance migrants. Oecologia. 2013;173:1101–12. 23660701 10.1007/s00442-013-2681-0

[63] Chernetsov N. Migratory stopovers of wrens *Troglodytes troglodytes* on the south-eastern baltic coast. Avian Ecol Beha. 2010;17:1–10.

[64] Arizaga J, Maggini I, Hama F, Crespo A, Gargallo G. Site- and species-specific fuel load of European–afrotropical passerines on arrival at three oases of southeast Morocco during spring migration. Bird Study. 2013;60:11–21. 10.1080/00063657.2012.735222

